# Charge mobility calculation of organic semiconductors without use of experimental single-crystal data

**DOI:** 10.1038/s41598-020-59238-2

**Published:** 2020-02-17

**Authors:** Hiroyuki Ishii, Shigeaki Obata, Naoyuki Niitsu, Shun Watanabe, Hitoshi Goto, Kenji Hirose, Nobuhiko Kobayashi, Toshihiro Okamoto, Jun Takeya

**Affiliations:** 1 Department of Applied Physics, Faculty of Pure and Applied Sciences, University of Tsukuba, 1-1-1 Tennodai, Tsukuba, Ibaraki 305-8573 Japan; 2 Educational Programs on Advanced Simulation Engineering, Toyohashi University of Technology, 1-1 Hibarigaoka, Tempaku-cho, Toyohashi, Aichi 441-8580 Japan; 3 CONFLEX Corporation, Shinagawa Center Bldg. 6F, 3-23-17 Takanawa, Minato-ku, Tokyo, 108-0074 Japan; 4 Material Innovation Research Center (MIRC) and Department of Advanced Materials Science, Graduate School of Frontier Sciences, The University of Tokyo, 5-1-5 Kashiwanoha, Kashiwa, Chiba, 277-8561 Japan; 5 JST, PRESTO, 4-1-8 Honcho, Kawaguchi, Saitama, 332-0012 Japan; 6 Department of Computer Science and Engineering, Toyohashi University of Technology, 1-1 Hibarigaoka, Tempaku-cho, Toyohashi, Aichi 441-8580 Japan; 7 International Center of Materials Nanoarchitectonics, National Institute for Materials Science (NIMS), 1-1 Namiki, Tsukuba, 305-0044 Japan

**Keywords:** Organic molecules in materials science, Electronic devices, Theory and computation, Two-dimensional materials, Electronic properties and materials

## Abstract

Prediction of material properties of newly designed molecules is a long-term goal in organic electronics. In general, it is a difficult problem, because the material properties are dominated by the unknown packing structure. We present a practical method to obtain charge transport properties of organic single crystals, without use of experimental single-crystal data. As a demonstration, we employ the promising molecule C_10_–DNBDT. We succeeded in quantitative evaluation of charge mobility of the single crystal using our quantum wave-packet dynamical simulation method. Here, the single-crystal data is computationally obtained by searching possible packing structures from structural formula of the molecule. We increase accuracy in identifying the actual crystal structure from suggested ones by using not only crystal energy but also similarity between calculated and experimental powder X-ray diffraction patterns. The proposed methodology can be a theoretical design technique for efficiently developing new high-performance organic semiconductors, since it can estimate the charge transport properties at early stage in the process of material development.

## Introduction

Prediction of material properties of newly designed molecules from their structural formulae is a long-term goal in organic electronics^1,2^. The efficiency of charge propagation is dominated by charge transfer between adjacent molecules^3,4^. In principle, in synthetic molecular design for high-performance organic semiconductors, in addition to the intramolecular *π*-conjugated structure, ideal intermolecular arrangement is required to maximize the charge transfer to enlarge the electronic density outside of the molecular frame^5^. However, even with enormous effort, the synthesis of new molecules does not lead to practically useful semiconductor materials in most cases because of poor charge carrier mobility due to non-ideal molecular packing, which is known only after crystallization of the synthesized molecules. It thus takes much time and effort to develop new high-performance organic semiconductors. Therefore, a new technique with help of theoretical approach is strongly desirable to estimate electronic properties of crystals of newly synthesized molecules at early stage in the development process to reduce time and effort.

Computational approaches to facilitate material developments, such as crystal structure prediction^6–10^ and material screening^11–16^, have much attention. In the field of organic electronics, application examples of crystal-structure and charge-transport-property predictions to candidate molecules have been recently reported^17–23^. The long-standing issue in study of crystal structure prediction is how to identify the observed polymorphs of a target molecule among a number of computationally suggested polymorphs. Most existing methods rank the predicted crystal structures based on their energies, which are calculated using dispersion-inclusive density functional theory (DFT)^8,24–29^. However, the experimentally observed structure does not always show the lowest energy since the structure strongly depends on the crystal-growth conditions, such as temperature, solvents, and film-forming methods. There is also an another practical problem that high computational cost of DFT based methods limits the molecular size and number of crystal structure candidates that can be evaluated.

As for prediction of charge transport properties of organic semiconductors, two conventional models are widely used, namely the incoherent hopping model^30,31^ and the coherent band-transport model^32^. However, these models cannot be applied to recent high-mobility organic semiconductors, because experimental studies, such as Hall effect^33–35^ and electron spin resonance measurements^36,37^, have revealed intermediate transport properties between the hopping and band limits. To overcome this problem, more rigorous theoretical approaches^38–42^ including reviews^43–46^ have been recently reported, but the quantitative prediction of the charge transport properties, including temperature dependence, of molecular crystals is still difficult even if accurate static crystal structures are obtained.

Here, we propose a practical method based on low-cost force-field and high-accuracy quantum simulations that can quantitatively estimate the charge transport properties of crystal structure of newly developed molecule from its structural formula using the non-perturbative approach based on quantum wave-packet dynamical simulations coupled with molecular mechanics, which enable us to access the intermediate regime between high-mobility band transport limit and low-mobility hopping transport limit. In the method, the crystal structure data is computationally obtained by searching possible packing structures from the structural formula. Furthermore, to identify the observed crystal structure with practically useful accuracy and low computational cost from a number of computationally suggested polymorphs, we use experimentally measured powder X-ray diffraction (PXRD) data, which has been used for the determination of the crystal structures^47–50^. The PXRD data are easily obtained after early-stage small-amount synthesis. Different from virtual screening approach for a large number of candidates^14,16^, our method combines computational and experimental approaches, and we aim to really create a novel and ideal semiconductor with examinations of a possibility of the synthesized molecule as high-performance semiconductor by the simulations with high-accuracy without a lot of experimental efforts. These theoretical supports can significantly reduce effort and time and provide useful information for the strategic design of single molecules in the development of high-performance semiconductors.

**Figure 1:**
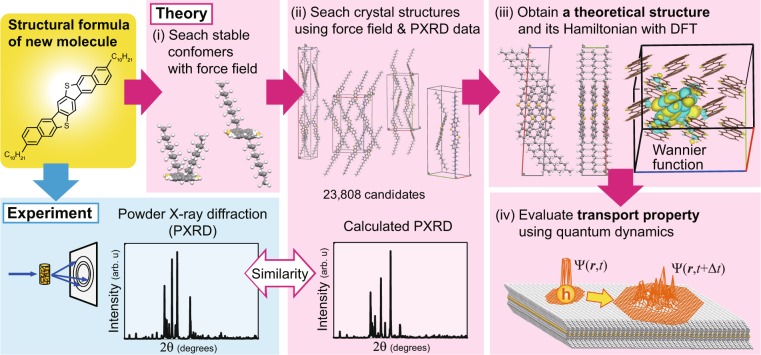
Schematic diagram of the proposed method for determining crystal structure and carrier transport properties of organic semiconductor. (**i**) Stable conformers of the target are obtained using force field calculations. (**ii**) Favorable crystal structures are selected from initial candidates based on force field calculations and ranked based on crystal energy and similarity between calculated and experimental PXRD patterns. (**iii**) Selected crystal structure according to the ranking is re-optimized based on quantum mechanics and theoretical crystal structure of the target is determined. (**iv**) Hole mobility of the theoretical crystal structure is evaluated using quantum dynamics.

Figure 1 shows a schematic diagram of the proposed method for determining crystal structure and carrier transport properties of newly developed molecule. In step (i), we search for energetically favorable conformers based on the structural formula of the target molecule using force field calculations. In step (ii), initial crystal structures are generated using each oriented molecule as the asymmetric unit and common space groups. Then, the initial crystal structures are optimized using molecular mechanics calculations with the force field. The rankings of favorable crystal structures are determined based on assessment value *A*_crystal_ derived from their calculated crystal energies and similarities between calculated and experimental PXRD patterns. In step (iii), the selected structure according to the ranking is re-optimized using quantum mechanical calculation based on DFT with dispersion correction (DFT-D), and the obtained structure is determined as theoretical crystal structure of the target. Finally, in step (iv), we evaluate charge transport properties of the theoretical crystal structure using order-*N* quantum dynamics calculations.

In the present work, we adopt Merck molecular force field 94 (MMFF94)^51^ as the force field and DFT-D with the Perdew-Burke-Ernzerhof parametrization of the generalized gradient approximation (GGA-PBE) exchange-correlation functional^52^ and the Tkatchenko-Scheffler (TS) scheme^53^ (PBE-TS). The similarity between PXRD paterns is calculated by using Gelder’s method^54^. Transport calculations are performed using the time-dependent wave packet diffusion (TD-WPD) method^41,55,56^, where the mobility of charge carriers coupled with phonons is evaluated in terms of a time-dependent expression of the Kubo formula using wave packet dynamics. We use 3,11-didecyldinaphtho[2,3-*d*:2′,3′-*d*’]benzo[1,2-*b*:4,5-*b*’]dithiophene (C_10_–DNBDT)^57^ as the practical target for demonstrating our approach and show the procedure for determining the crystal structure and associated carrier transport performance. Since single-crystal structure and charge transport property of C_10_–DNBDT have been well investigated experimentally^58^, C_10_–DNBDT is one of the best choice for validation of our approach.

## Results and Discussion

### Crystal structure determination

In step (i), we found two energetically stable conformers with *C*i or *C*_2_ point group symmetries of the C_10_–DNBDT molecule using the MMFF94 potential (Supplementary Fig. S1). We employed the conformer with the *C*i symmetry for the generation of initial crystal structures because known semiconductor molecules usually show a conformation with inversion symmetry in their crystal structure^59,60^. It is considered that for such molecules, this conformation has the advantages of close molecular packing and energetic stability.

In step (ii), 23,808 initial crystal structures were generated using single molecules with different orientations and nine common space groups without inversion symmetry in the Cambridge Structural Database (CSD)^61^. After the optimization of all initial crystal structures using the MMFF94 potential, we found 348 unique structures within 5.0 kcal/mol of the global minimum in crystal energy. Then, in order to identify the observed crystal structure, the unique structures were ranked based on their assessment values *A*_crystal_ derived from the crystal energy *E*_crystal_ and *S*_PXRD_ (Table 1 and Supplementary Table SI). *S*_PXRD_ shows the similarity between the calculated PXRD pattern of a crystal structure and the experimentally observed PXRD pattern. In the presented method, we assess that the crystal structure with the lowest *A*_crystal_ value is most similar to the observed crystal structure. According to the ranking based on *A*_crystal_, the 1st structure was determined as the observed crystal structure of C_10_–DNBDT in the crystal structure search using MMFF94 potential and was employed for the next step. Here, as references, we show top 4 structures in Table 1 and Supplementary Fig. S3 and discuss them in the supplement. Table 1 shows that we can increase accuracy in identifying the actual crystal structure from computationally suggested structures by using both the crystal energy and PXRD pattern similarity, since the 1st structure has the rank of 25th in the crystal energy and 29th in PXRD pattern similarity. The crystal energy landscapes and assessment value landscapes for C_10_–DNBDT are shown in the supplement to summarize the results of the crystal structure search (Supplementary Fig. S2).

**Table 1:** Comparison of computational crystal structures with the experimental structure.

Crystal structure	Space group	*a*/Å (Δ*a*/%)	*b*/Å (Δ*b*/%)	*c*/Å (Δ*c*/%)	*β*/° (Δ*β*/%)	*θ*_tor_/° (Δ*θ*_tor_/%)	*θ*_her_/° (Δ*θ*_her_/%)	RMSD_20_^*c*^/Å	*A*_crystal_/kcal mol ^−1^	*E*_crystal_/kcal mol ^−1^	*S*_PXRD_
1st	*P*2_1_/*c*	37.879	7.354	7.048	99.31	54.89, −54.89	29.40	1.123	3.738 (1)	18.982 (25)	0.932 (29)
2nd	*C*2/*c*	74.952	7.356	7.048	94.02	54.87, −54.87	29.43	1.218	3.745 (2)	18.994 (26)	0.932 (28)
3rd	*P*2_1_2_1_2_1_	7.385	74.836	7.061	90	55.03, −54.76	29.77	4.959	3.997 (3)	19.447 (56)	0.940 (8)
4th	*P*2_1_2_1_2	7.388	74.733	7.056	90	54.90, −55.08	29.86	4.926	4.037 (4)	19.342 (48)	0.934 (24)
Theory^*a*^	*P*2_1_/*c*	39.134 (−2.3)	7.239 (−7.4)	6.349 (3.9)	96.30 (2.1)	59.34, −59.34 (−6.2, 6.2)	38.36 (−20.6)	0.584	—	—	0.984
Expt.^*b*^	*P*2_1_/*c*	40.039	7.818	6.112	94.36	63.27, −63.27	48.33	0.000	—	—	0.995

Space group, lattice parameters, RMSD_20_, assessment value *A*_crystal_, crystal energy *E*_crystal_, and PXRD pattern similarity *S*_PXRD_ are listed for each structure. Numbers in parenthesis in column of *A*_crystal_, *E*_crystal_ and *S*_PXRD_ represent the rank based on only *A*_crystal_, *E*_crystal_ and *S*_PXRD_, respectively. The 1st to 4th crystal structures are the obtained ones in the force field (MMFF94) calculations. ^*a*^The theoretical crystal structure is obtained by optimization of the 1st structure using DFT-D (PBE-TS). ^*b*^The experimental structure was obtained at 298 K. ^*c*^For the 3rd and 4th structures, clusters of 20 molecules could not be overlaid with the experimental structure due to a large structure difference; therefore, their RMSD_16_ values are shown.

**Figure 2:**
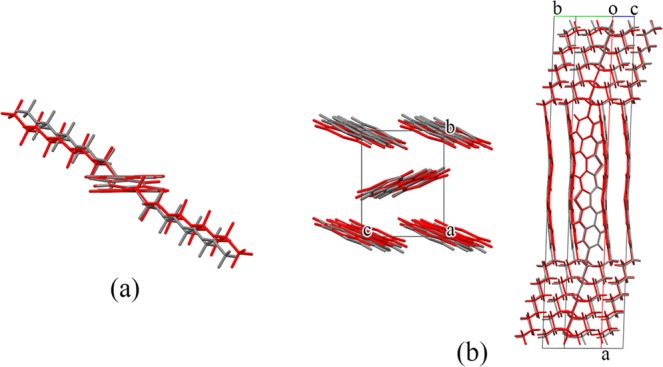
Superposition of theoretical (red) and experimental (gray) crystal structures of C_10_–DNBDT. (**a**) Conformation in the crystal structure and (**b**) molecular packing. The theoretical structure was obtained by re-optimizing the 1st structure in Table 1 using DFT-D based on the PBE-TS scheme. For visibility, alkyl side chains are omitted in the left-hand-side image in (**b**).

In step (iii), the 1st structure was re-optimized using DFT-D based on the PBE-TS scheme, and the obtained structure was determined as the theoretical crystal structure of C_10_–DNBDT. In Fig. 2, we superpose the theoretically and experimentally determined crystal and molecular structures. The molecular conformation and packing arrangements in the theoretical crystal structure are in good agreement with those of the experimental crystal structure. These results demonstrate that the proposed method can be used to detect the actual crystal structure of the C_10_–DNBDT molecule.

We next discuss the quantitative difference between the theoretical and experimental crystal structures in terms of Δ*θ*_tor_, Δ*θ*_her_, and RMSD_20_ in Table 1. *θ*_tor_ is the torsion angle around bonds between the *π*-conjugated core and the alkyl group, and *θ*_her_ is the angle between planes defined by *π*-conjugated cores of C_10_–DNBDT molecules related by the 2-fold screw axis symmetry in the layer (Supplementary Fig. S3). RMSD_20_ is the root-mean-square deviation in atomic positions, ignoring H atoms, between matching clusters of 20 molecules from the theoretical and experimental crystal structures^62^. These values were calculated using the software Mercury^63^. The molecular conformation and the herringbone arrangements in the theoretical structure reproduce these features in the experimental structure, with an absolute Δ*θ*_tor_ value of 6.2% and a Δ*θ*_her_ value of −20.6%. The atomic positions in the theoretical structure match those in the experimental structure, with an RMSD_20_ of 0.584 Å. In addition, the *S*_PXRD_ value of the theoretical crystal structure is 0.984 (Table 1). This result indicates that the calculated and experimental PXRD patterns match very well. The effects of the calculation method on the representation accuracy of the experimental structure, which are important for the accuracy of computational determinations for crystal structures and physical properties, are described in the supplement (Supplementary Table SII). Although the MMFF94 and PBE-TS work for estimating C_10_–DNBDT, we agree possibility of existence of more adequate selection of force fields and DFT functionals for organic semiconductors. The selection should be updated as the computational technology advances, from a viewpoint of accuracy and cost.

### Transport property evaluation

In step (iv), we evaluate the hole mobility of a C_10_–DNBDT single crystal using the theoretical crystal structure. A quantum dynamical approach called TD-WPD method^56^ is used for this task, where the mobility is evaluated using a time-dependent expression of the Kubo formula. Intermolecular vibrations at around room temperature induce a large dynamic disorder in the transfer integrals and become a major carrier scattering source. Therefore, the effects of the interaction between charge carriers and molecular vibrations on transport properties are introduced as time-dependent transfer integrals. To obtain accurate intermolecular transfer integrals, we evaluate the transfer integrals using DFT-D with maximally localized Wannier functions (MLWFs)^64,65^. The intermolecuar vibrational modes can be obtained with practically useful accuracy based on low-cost force field calculations using the software CONFLEX^66^. The details of the procedure used for the computation of transport properties are described in the methods section and supplement.

**Figure 3:**
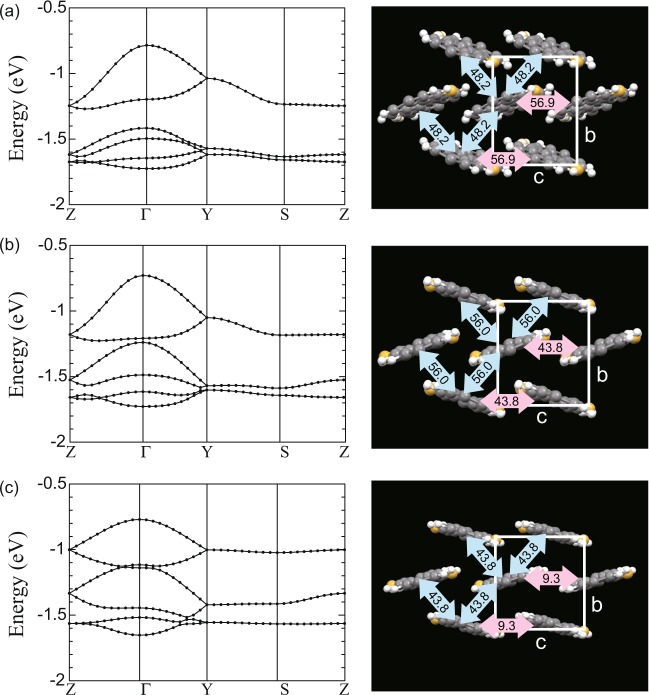
Calculated band structures and intermolecular transfer integrals. (**a**) HOMO band dispersions and intermolecular transfer integrals (meV) of the experimental structure obtained at 298 K, (**b**) those of the theoretical structure obtained using the PBE-TS scheme, and (**c**) those of the 1st structure obtained using MMFF94. The band dispersions are drawn with symmetry points of *Z*(0,0,1/2), Γ(0,0,0), *Y*(0,1/2,0), and *S*(0,1/2,1/2). For visibility, alkyl side chains are omitted in the right-hand-side figures.

Before calculating the transport properties, we evaluate the electronic states. A C_10_–DNBDT single crystal is a p-type organic semiconductor, and thus its transport properties are characterized by the band structure originating from the highest occupied molecular orbitals (HOMOs). Figure 3(a–c) show the calculated HOMO band dispersions (left) and intermolecular transfer integrals (right) of the experimental structure obtained at 298 K, those of the theoretical structure, and those of the 1st structure in Table 1, respectively. The intermolecular transfer integrals were obtained as Hamiltonian matrix elements on the MLWF basis set. Because C_10_–DNBDT single crystals with a layered structure are regarded as two-dimensional electron systems, the band dispersions and the transfer integrals on the *bc* plane are calculated. The transfer integrals of the theoretical structure, shown in Fig. 3(b), are very close to those of the experimental structure, shown in Fig. 3(a), with an accuracy of about 10 meV. The relatively low reproducibility with respect to *θ*_her_ of the theoretical crystal structure is ascribed to the slight difference in transfer integrals (Table 1). A comparison of Fig. 3(b,c) reveals that the PBE-TS re-optimization of the 1st structure dramatically improved the red-colored transfer integrals, from 9.3 meV to 43.8 meV, making them similar to those of the experimental structure (56.9 meV). This improvement is due to the lattice constant *c* of 7.048 Å for the 1st structure being overestimated compared to that of 6.112 Å for the experimental structure.

**Figure 4:**
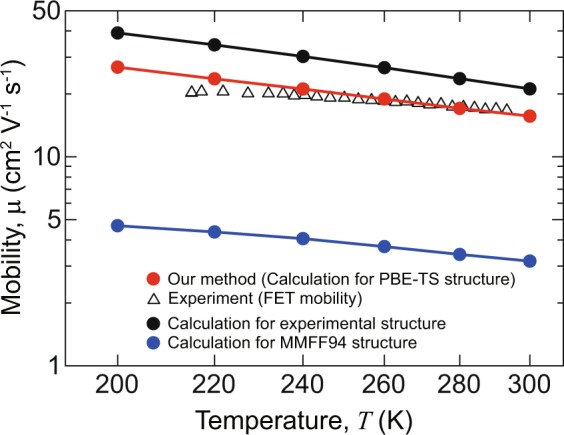
Temperature dependence of mobility along the column direction in a C _10_–DNBDT single crystal. Black, red, and blue circles represent the calculated mobilities for the experimental structure, the theoretical structure obtained using PBE-TS, and the 1st structure obtained using MMFF94, respectively. For comparison, the experimental values of FET mobilities^58^ are also shown as triangles.

Using the obtained transfer integrals, we estimate the intrinsic mobility along the column direction (*c*-axis) of a C_10_–DNBDT single crystal. First, we compare the estimated mobility using the theoretical crystal structure with the experimentally observed mobility of a single-crystal field-effect transistor (FET)^58^, shown by red circles and triangles in Fig. 4, respectively. The estimated mobilities, which are 15.7 cm^2^ V^−1^ s^−1^ at 300 K and 23.6 cm^2^ V^−1^ s^−1^ at 220 K, show good agreement with the FET mobilities, which are 17 cm^2^ V^−1^ s^−1^ at 290 K and 20 cm^2^ V^−1^ s^−1^ at 220 K. The carrier transport exhibits band-like transport, with an increase in mobility with decreasing temperature. This implies that the wavefunctions of holes in a C_10_–DNBDT single crystal spread over the molecules, leading to coherent band-like transport. The discrepancy between the estimated mobilities and the FET mobilities weakly increases with decreasing temperature. This is because actual organic single crystals inevitably have static disorder, caused by defects and impurities, which becomes another carrier scattering source different from molecular vibrations. In contrast, to estimate the intrinsic transport properties, our simulations take only the interaction between electrons and molecular vibrations into account.

To remove the static disorder effects from the experimentally observed mobility, we calculated the mobility using the experimental structure. The temperature dependence is shown as black circles in Fig. 4. The calculation results imply that the experimentally observed FET mobility (shown as triangles) has the potential to improve up to the values shown as black circles when static disorder is reduced. Furthermore, the slight difference between the red and black circles confirms the sufficient accuracy of the theoretical crystal structure in terms of transport properties. The mobility calculated from the 1st structure (blue circles) becomes much lower than the estimated mobility of the theoretical crystal structure due to the low reproducibility of herringbone arrangements in the force field calculation (Table 1). Therefore, we can conclude that PBE-TS re-optimization is required to increase the accuracy of the theoretically estimated mobility.

### Computational and experimental cost

The proposed methodology can detect crystal structure and intrinsic transport properties from structural formula and measured PXRD data of a target molecule usually within 1–3 months. The calculation times for steps (i) to (iv) in Fig. 1 for the target molecule considered here are as follows. It took 22 days to find energetically favorable crystal structures using parallel computing with 200 cores. The crystal structure search scales almost linearly with the number of CPU cores^67^; for example, when using 400 cores, the calculation time can be shortened by about half to about 10 days. The experiments to measure the PXRD pattern can be progressed simultaneously with the theoretical crystal structure search. In many cases, the synthesis, creation of powder crystal and measurement of PXRD data for the target molecule require 1–30, 1–90, and ≤1 days, respectively. Re-optimization using DFT-D required 15 days using parallel computing with 8 cores. The calculation time required to evaluate the transport properties at a given temperature was about 4.5 days, including the generation of material parameters, such as intermolecular transfer integrals and coupling constants between electron and molecular vibrations, using parallel computing with 20–80 cores. Detailed information about the computation times and resources for each calculation procedure is given in the supplement (Supplementary Table SIII). In general, much more time and effort are required to develop practically useful semiconductor material using only experiments because intrinsic charge transport properties of newly developed material are not clear until a experimental measurement for the single crystal is performed and, furthermore, the observed performance is not always high. By applying the proposed methodology to the development of new semiconductor molecules, candidate molecules that theoretically have high performance can be efficiently found at early stage in the process of material development, significantly reducing the experimental effort and accelerating the development process. In order to establish more high-efficient methodology, we should further improve the computational cost by applying high-throughput simulation techniques to our methodology.

## Summary

We proposed a method for determining the crystal structure of organic molecule and associated transport properties without use of experimental single-crystal structure data. First, we find energetically stable conformers from the structural formula of the target molecule and obtain favorable crystal structures from the initial candidates based on force field calculations. Second, based on the experimentally observed PXRD pattern and calculated crystal energy, possible crystal structure is selected. Third, theoretical crystal structure of the target is determined by re-optimization using DFT-D based on quantum mechanics. Finally, we evaluate the hole mobility for the theoretical crystal structure via quantum dynamics using the TD-WPD method, where the transport properties can be evaluated by the Kubo formula using the wave packet dynamics of charge carriers interacting with molecular vibrations. As a demonstration, the method was applied to the C_10_–DNBDT molecule, and the crystal structure and its transport properties were determined. Crystal structure candidates were narrowed down from 23,808 initial candidates to 348 using molecular mechanics, and to 1 using PXRD data and quantum mechanics calculation. The theoretical crystal structure and transport properties show good agreement with experimental results. Although we should further benchmark our methodology by using other organic semiconductors to elucidate a usefulness and transferable ability, we showed that our methodology can detect the crystal structure and intrinsic transport properties of practical organic semiconductor molecule from structural formula and measured PXRD data on a realistic timescale with useful accuracy. The strategy in our methodology is available for other targets with structural formula and PXRD data and is expected to be useful for developing new high-performance semiconductor materials.

## Methods

### Procedures of crystal structure determination

The crystal structure determination of an organic molecule is performed in three steps, corresponding to steps (i)–(iii) in Fig. 1. In the present work, the target molecule for practical demonstration was C_10_–DNBDT. The calculations for finding and assessing crystal structures in steps (i) and (ii) are performed using our original method^8,67^ and MMFF94^51^ implemented in the software CONFLEX^66^. We employ DFT-D with the GGA-PBE exchange-correlation functional^52^, on-the-fly ultrasoft pseudopotential, and TS scheme^53^. Refinement of the crystal structure using DFT-D based on the PBE-TS scheme in step (iii) is executed using the software CASTEP^68^ in Materials Studio 2017^69^. The MMFF94 and PBE-TS have been used for our previous studies and have provided a good accuracy for structure reproducibility and relative stability of crystals^8,67,70^.

In step (i), to detect a stable conformation of alkyl groups with an all-trans conformation relative to the *π*-conjugated core in C_10_–DNBDT, we perform a conformational search by changing the torsion angles around bonds between the *π*-conjugated core and the alkyl groups in steps of 10 degrees. Here, we employ the all-trans as initial conformation of alkyl groups because the conformation is thought to provide a high density packing as observed in known crystal structures^59,60^.

In step (ii), one conformer obtained from the previous step is selected and rotated around the *x*, *y*, and *z* axes in steps of 30 degrees. The number of molecules in the asymmetric unit is set to one, and nine common space groups without inversion symmetry are employed, namely *P*1, *P*2_1_, *C*2, *Pc*, *Cc*, *P*2_1_2_1_2, *P*2_1_2_1_2_1_, *Pca*2_1_, and *Pna*2_1_, considering the inversion symmetry of C _10_–DNBDT (Supplementary Fig. S1) and space group frequency ranking using the CSD^61^. The initial crystal structures are generated using each oriented molecule as the asymmetric unit and the common space groups as well as some of molecular positions and different lattice constant parameters. Then, we optimize intramolecular geometry, molecular orientation and translation, and unit cell dimensions in each initial crystal structure by minimizing crystal energy *E*_crystal_ under the specified space group symmetry. Thus, in the optimization, we employ a fully flexible molecular model and the initial molecular conformation including the alkyl groups changes to adequate conformation for packing arrangements due to intermolecular interactions. For all optimized crystal structures, it is confirmed that they have no imaginary frequencies by performing a normal mode analysis in the atomic coordinates under the specified space group symmetry.

Next, unique crystal structures within 5.0 kcal/mol of the global minimum in crystal energy are extracted from all optimized structures because the observed crystal structures were nearly always found within 5.0 kcal/mol in previous studies^8,70,71^. Here, we introduce an assessment value calculated as *A*_crystal_ = Δ*E*_crystal_ + *α*Δ*S*_PXRD_, where Δ*E*_crystal_ is the crystal energy difference from the global minimum, and Δ*S*_PXRD_ is obtained by subtracting *S*_PXRD_ from 1.0. *S*_PXRD_ represents the similarity between the calculated and experimental PXRD patterns and is calculated by using Gelder’s method^54^. With *α* set to 25 kcal/mol, the discrepancy between the calculated and observed structures defined by a Δ*S*_PXRD_ value of 0.2 is estimated as an energetic penalty of 5.0 kcal/mol in the assessment of possible crystal structures. The parameter *α* should be adjusted if we change the force field because the effective range of relative energy between polymorphs depends on the force field. The rankings of the unique crystal structures are decided based on their assessment values, *A*_crystal_; the top four structures for the target molecule in this study are shown in Table 1. The details of the procedure in step (ii) are described in the supplement.

In step (iii), the crystal structure, which is selected according to the ranking, is re-optimized using DFT-D with the PBE-TS scheme. For the re-optimization of 1st structure in this work, the cutoff energy for the plane wave and the total energy convergence tolerance are set to 450 eV and 0.5 × 10^−5^ eV/atom, respectively, and the Brillouin zone integration is performed with a 1 × 3 × 4 *k*-point set.

### Calculations of carrier mobility

We evaluate the intrinsic hole mobility of a C_10_–DNBDT single crystal using our order-*N* quantum dynamics simulation technique called TD-WPD^56^. The charge transport properties are characterized by the intermolecular transfer integrals, the molecular vibrations, and the coupling between holes and molecular vibrations. The transfer integrals between molecules are computed using the high-accuracy Wannier method^64,65^ based on DFT-D^72^. We employ the 1 × 2 × 3 super-cell structure. The cutoff energies for the plane wave and charge density are 80 and 800 Ry, respectively. Brillouin zone integration is performed at the Γ point. The normal modes of molecular vibrations are obtained using force field calculations with low computational cost using the software CONFLEX^66^. The magnitudes of coupling constants are evaluated from the change in transfer integrals due to atomic displacement along the normal mode. We employ a monolayer consisting of 200 × 200 unit cells to obtain the trajectory of wave packets. The wave packet dynamics are computed up to 2 ps with a time step of 0.5 fs. The details of the procedure for the computation are described in the supplement.

### Acquisition of experimental reference data for the crystal structure determination

First, we describe the acquisition of experimental PXRD data, which are used for the crystal structure determination. In the preparation of powder samples, *o*DCB solution of C_10_–DNBDT (2 mg mL^−1^) was slowly cooled from 110 °C to room temperature for 10 h. The resulting crystals were collected by filtration and dried in vacuo at 140 °C. PXRD measurement of C_10_–DNBDT was performed using synchrotron light source with a wavelength of 1.08 Å at BL44B2 of SPring-8 (Proposal 20160029). Microcrystalline powder samples in capillary tube with a diameter of 0.3 mm were used for transmission method with a exposure time of 210 seconds. Diffraction data were collected on an imaging plate.

Second, we describe the acquisition of experimental crystal structure data of C_10_–DNBDT. In order to validate the theoretical crystal structure determined by the proposed methodology and the measured PXRD data, we perform single-crystal X-ray diffraction (XRD) measurement and determine the crystal structure. Single crystals were obtained by means of recrystallization from certain organic solvents. Those C_10_–DNBDT (10 mg mL^−1^) were grown via the gradual cooling of *o*-dichlorobenzene (*o*DCB) solution from 120 °C to room temperature for 24 h. The prepared crystals were collected and dried on filter paper. The XRD data for the single crystals of C_10_–DNBDT were collected on a Rigaku XtaLAB Synergy imaging plate diffractometer with Cu K *α* radiation.

### Device fabrication and characterization of C_10_–DNBDT FETs

A device was fabricated on a polyethylene naphthalate (PEN) substrate. The PEN (Teijin, Q65HA-125) film was prebaked at 150 °C for three hours in a vacuum oven to reduce thermal expansion, and then cleaned with acetone and 2-propanol in an ultrasonic bath. A 50 nm thick layer of gold as the gate electrode was deposited using thermal evaporation through a shadow mask. As a gate dielectric, a fluorinated polymeric insulator (EPRIMA AL-X601, Asahi Glass Co.) was used. A single crystal of C_10_–DNBDT was then grown on top of the gate dielectric layer from purified 3-chlorothiophene (Pi-Crystal Inc.) with a concentration of 0.025 wt% via continuous edge casting at 110 °C. A gold source and drain electrodes were thermally evaporated through a shadow mask. Finally, laser etching was performed (V-Technology Co., Ltd., Callisto (266 nm)) for the C_10_–DNBDT layer to form a precise Hall bar architecture. The channel length (*L*) and width (*W*) were 200 *μ*m and 50 *μ*m, respectively. Four probes were mounted between the source and drain electrodes, where the distance between two longitudinal probes along the channel length (defined as L*) was designed to be 75 *μ*m. The temperature *T* dependence of the four-terminal mobility measurements was evaluated in a He gas-exchange cryostat. The transfer characteristics with *V*_D_ = −2 V were continuously recorded by sweeping *V*_G_ during the slow ramping of *T* down and up, where the *T* ramping rate (0.2 K min^−1^) was much slower than the rate for *V*_G_ scans (approximately 1 V s^−1^).

## Supplementary information

Supplementary Information.
