# Supplementary material for: Charge mobility calculation of organic semiconductors without use of experimental single-crystal data
Source: Sci Rep. 2020 Feb 17;10:2524. doi: 10.1038/s41598-020-59238-2 (PMC7026405; doi:10.1038/s41598-020-59238-2)
Supplement: Supplementary file 1 — Supplementary Information. [file 41598_2020_59238_MOESM1_ESM.pdf]

Supplementary Information for

Charge mobility calculation of organic semiconductors without use of experimental single-crystal data

Hiroyuki Ishii*, Shigeaki Obata*, Naoyuki Niitsu, Shun Watanabe, Hitoshi Goto, Kenji Hirose, Toshihiro Okamoto, and Jun Takeya

Correspondence and requests for materials should be addressed to ishii@bk.tsukuba.ac.jp (H.I.), and obata@conflex.co.jp (S.O.)

This PDF file includes:

1. Structure configuration
2. Crystal-structure-search methodology
3. Transport-calculation methodology
4. Computational resources and times

Figures S1, S2, S3 and S4

Tables SI, SII, and SIII

1. Structure configuration

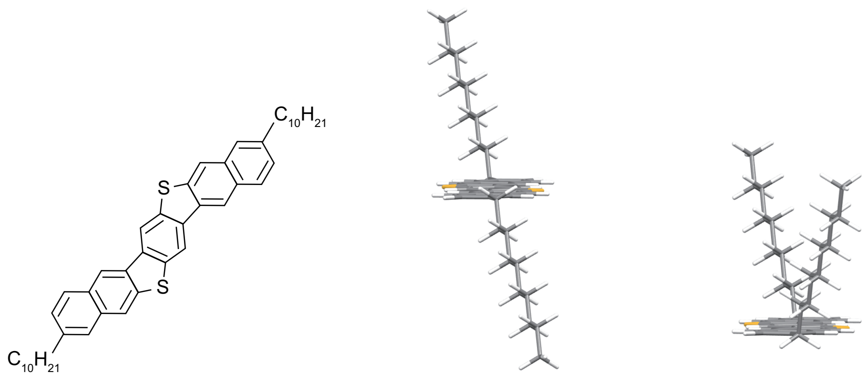

(a) (b) (c)

SI Fig. S1. **Structural formula of C_{10}–DNBDT molecule and conformations based on MMFF94 potential.** (a) Structural formula of target molecule, C_{10}–DNBDT. (b) Conformation with C_i point group symmetry, (c) Conformation with C_2 point group symmetry.

Figure S1 shows the structural formula of the C_{10}–DNBDT molecule and its two conformers obtained from a conformational search based on the MMFF94 potential. We considered a reasonable assumption that C_{10}–DNBDT has a conformation with inversion symmetry in its crystal structure, because known semiconductor molecules usually show a conformation with inversion symmetry in their crystal structure [1, 2]. Thus, we employed the conformer with the Ci point group symmetry for the generation of initial crystal structures.

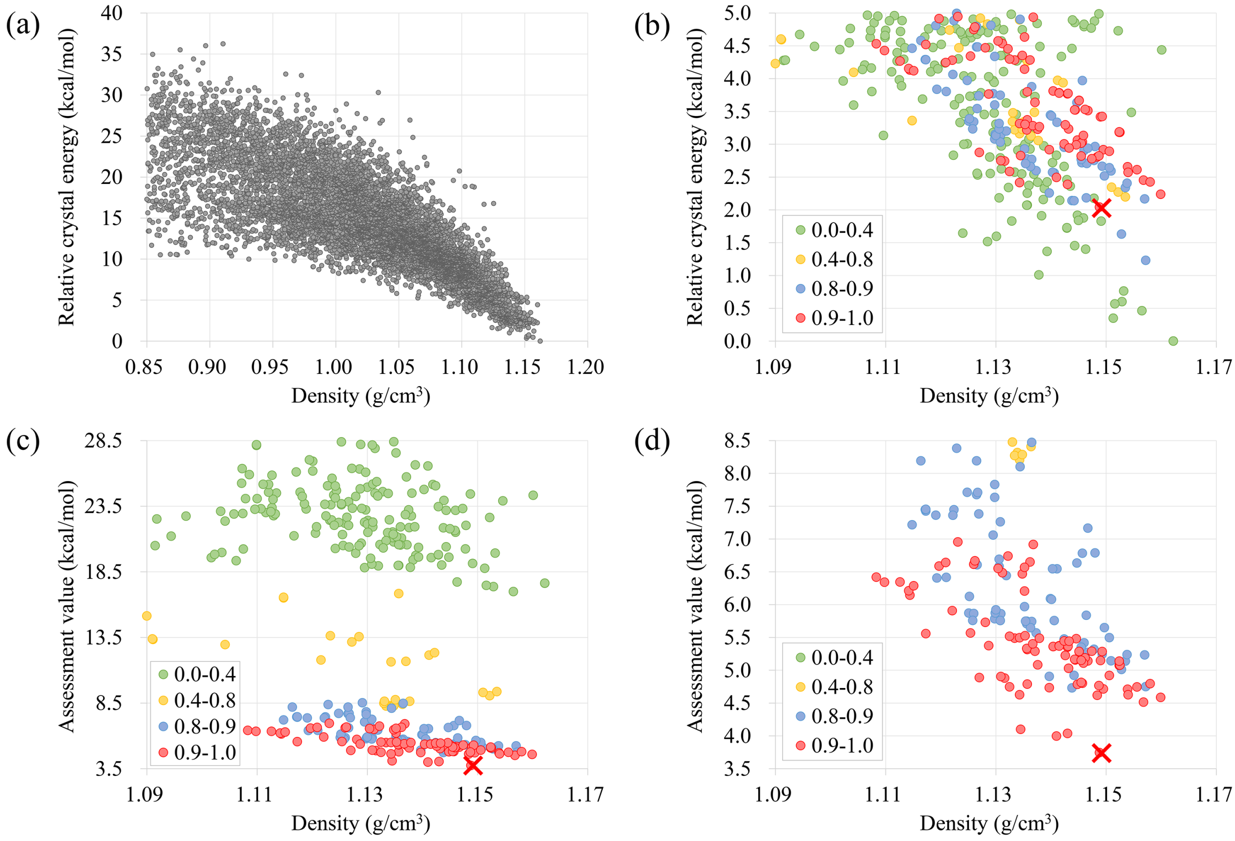

SI Fig. S2. **Crystal-energy landscapes (a, b) and assessment-value landscapes (c, d) for C_{10}–DNBDT.** Each symbol represents one crystal structure. The color coding of symbols in (b), (c), and (d) are defined according to PXRD pattern similarity value. The cross marks in (b), (c), and (d) indicate the structure matching the known crystal of C_{10}–DNBDT and the structure has the lowest A_{crystal} value.

Figures S2 (a) and (b) show crystal energy landscapes for C_{10}–DNBDT by plotting the relative crystal energy against the density of each structure obtained from the crystal structure search. The crystal energy landscape shows a typical trend which higher density packing has lower crystal energy (Fig. S2 (a)). The crystal structure search could find the structure corresponding to the known crystal of C_{10}–DNBDT (the cross-mark in Fig. S2 (b)), and the structure shows 25th and 2.03 kcal/mol above the global minimum in the crystal energy (Tab. SI and Fig. S2 (b)). Most structures with lower crystal energy than the structure matching the known crystal have a layered structure with interdigitated alkyl chains.

Here, we focus on subtle difference between the structures with 1st and 25th in the crystal energy. The lowest crystal energy among unique crystal structures obtained in the

crystal structure search was 16.953 kcal/mol on MMFF94 potential. The crystal with the lowest crystal energy, $E_{\mathrm{crystal}}(1)$, has the layered structure with interdigitated alkyl chains and shows $P2_1/c$ space group, a = 26.386 Å, b = 7.988 Å, c = 9.120 Å, and β = 94.79 °. The crystal structure with $E_{\mathrm{crystal}}(1)$ was re-optimized by PBE-TS method, results in a = 25.053 Å, b = 7.855 Å, c = 9.150 Å, and β = 91.87 °. In the re-optimization, the cutoff energy for the plane wave and the total energy convergence tolerance are set to 450 eV and 0.5×10^{-5} eV/atom, respectively, and the Brillouin zone integration is performed with a $1 \times 3 \times 3$ k-point set. The energy difference calculated by subtracting $E_{\mathrm{crystal}}(1)$ from $E_{\mathrm{crystal}}(25)$ is 2.03 and 2.05 kcal/mol on MMFF94 and PBE-TS calculations, respectively. Therefore, the theoretical analysis indicates that the layered structure with interdigitated alkyl chains, which is not observed in the known crystal of C_{10}–DNBDT, is energetically more stable. The conventional method by using relative energies based on dispersion-inclusive DFT calculation for detecting the actual crystal structure among a number of theoretically suggested polymorphs is thought to difficultly apply to the blind prediction of common semiconductor molecules consisting of π-conjugated core and alkyl groups such as C_{10}–DNBDT. The assessment by PXRD pattern similarity is helpful for detecting the actual crystal structures.

On the other hand, the theoretically determined structure always shows errors in the lattice parameters from the experimental structure, while PXRD pattern is sensitive to the parameters. Low quality of measured PXRD data as well as the errors in the lattice parameters make it difficult to detect the actual crystal structures from candidate structures by PXRD pattern similarity. Thus, we considered that the assessment of A_{crystal} by using both the relative energy and PXRD pattern similarity is reasonable for realizing high reliability in the detection of actual crystal structures.

Figures S2 (c) and (d) show assessment value landscapes for C_{10}–DNBDT by plotting the assessment value, A_{crystal}, against the density of each structure within 5.0 kcal/mol of the global minimum in the crystal energy. Table SI shows the top 10 crystal structures of C_{10}–DNBDT in the A_{crystal} ranking, and the structural differences between the top 4 structures are described in detail below (Fig. S3). The symbols in the assessment value landscape are arranged depending on PXRD pattern similarity (Figs. S2 (c) and (d)) unlike the crystal energy landscape (Fig. S2 (b)). The structures that have higher PXRD pattern similarity, that is, are similar to the observed crystal structure of C_{10}–DNBDT show lower A_{crystal} value

(Figs. S2 (c) and (d), and Fig. S3). On the other hand, the structures with low similarity to the observed crystal structure have higher A_{crystal} value and can be easily excluded from candidates of computationally suggested structure as the observed crystal (Fig. S2 (c)). In the assessment of possible crystal structures by A_{crystal} (Figs. S2 (c) and (d)), the crystal structure with the lowest A_{crystal} value is most similar to the observed crystal structure according to the theoretical assessment of energetic stability and structural similarity by the crystal energy and PXRD pattern similarity. Therefore, the 1st structure in the A_{crystal} ranking was determined as the observed crystal structure of C_{10}–DNBDT in the crystal structure search using MMFF94 potential. The atomic positions in the 1st structure match those in the experimental structure, with an RMSD_{20} of 1.123 Å (Tab. SII).

Final rank	A_{crystal} /kcal mol^{-1}	E_{crystal} (Rank) /kcal mol^{-1}	S_{PXRD} (Rank)	Space group	Z	Density /g cm^{-3}	a/Å	b/Å	c/Å	β/°
1	3.738	18.982 (25)	0.932 (29)	$P2_1/c$	2	1.150	37.879	7.354	7.048	99.31
2	3.745	18.994 (26)	0.932 (28)	$C2/c$	4	1.150	74.952	7.356	7.048	94.02
3	3.997	19.447 (56)	0.940 (8)	$P2_12_12_1$	4	1.142	7.385	74.836	7.061	90
4	4.037	19.342 (48)	0.934 (24)	$P2_12_12$	4	1.144	7.388	74.733	7.056	90
5	4.100	19.781 (94)	0.949 (6)	$Pbca$	4	1.136	7.353	7.072	75.482	90
6	4.514	19.406 (54)	0.918 (60)	$P2_12_12_1$	4	1.158	4.322	6.091	146.193	90
7	4.587	19.190 (38)	0.906 (76)	$P2_1/c$	2	1.161	6.116	4.309	72.856	91.44
8	4.619	19.759 (91)	0.927 (36)	$P2_12_12$	4	1.150	147.026	4.317	6.109	90
9	4.626	19.525 (64)	0.918 (58)	$C2$	4	1.155	146.853	4.311	6.096	88.71
10	4.626	19.370 (50)	0.912 (71)	$P2_1/c$	2	1.135	5.107	72.997	5.274	93.18

SI Tab. SI. **Top 10 crystal structures of C_{10}–DNBDT obtained in the crystal structure search.**

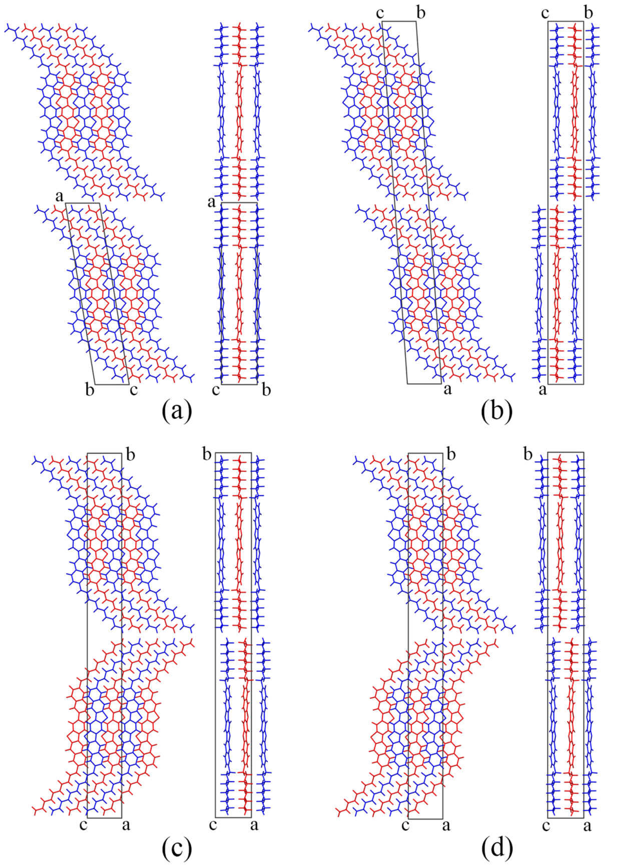

SI Fig. S3. **Top 4 crystal structures of C_{10}–DNBDT.** (a) 1st, (b) 2nd, (c) 3rd, and (d) 4th structures in Table 1. The red and blue molecules in a given layer in each structure are related by 2-fold screw axis symmetry.

Figure S3 shows the top 4 crystal structures in Table 1. The four crystal structures have a layered structure, as shown in Figure S3. The 1st, 2nd, 3rd, and 4th structures have space groups $P2_1/c$, $C2/c$, $P2_12_12_1$, and $P2_12_12$, respectively (Tab. SI). The layers in all four crystal structures have identical herringbone molecular arrangements, formed by 2-fold screw axis symmetry, but the stacking relationship between layers is different for each structure. The 1st and 2nd structures create a layered structure via the translational symmetry of the lattice and the C-centering symmetry of the $C2/c$ space group, respectively. The stacked layer along the a-axis in the 2nd structure is shifted by 0.5 along the b-axis and by 0.5 along the c-axis compared with that for the 1st structure (Figs. S3 (a) and (b)). The layers in the 3rd and 4th structures are related by the 2-fold screw axis with direction [0, 0, 1] at 1/4, 0, z with screw component [0, 0, 1/2] and by the 2-fold rotation axis with direction [0, 0, 1] at

0, 0, z, respectively. The stacked layer along the b-axis in the 3rd structure is shifted by 0.5 along the c-axis compared with that for the 4th structure (left-hand-side images in Figs. S3 (c) and (d)).

Structure	Density/g cm^{-3} (ΔD/%)	a/Å (Δa/%)	b/Å (Δb/%)	c/Å (Δc/%)	β/° ($\Delta\beta$/%)	θ_{tor}/° ($\Delta\theta_{tor}$/%)	θ_{her}/° ($\Delta\theta_{her}$/%)	$RMSD_{20}$ /Å
Experiment	1.168	40.039	7.818	6.112	94.36	63.27	48.33	0.000
PBE-TS	1.247	39.134	7.239	6.349	96.30	59.34	38.36	0.584
	(6.7)	(-2.3)	(-7.4)	(3.9)	(2.1)	(-6.2)	(-20.6)	
PBE-Grimme	1.298	37.683	7.034	6.551	98.51	49.66	36.25	1.403
	(11.1)	(-5.9)	(-10.0)	(7.2)	(4.4)	(-21.5)	(-25.0)	
PW91-OBS	1.624	35.650	6.108	6.512	104.60	35.47	19.87	2.464
	(39.0)	(-11.0)	(-21.9)	(6.6)	(10.9)	(-43.9)	(-58.9)	
MMFF94	1.150	37.879	7.354	7.048	99.31	54.89	29.40	1.123
	(-1.6)	(-5.4)	(-5.9)	(15.3)	(5.2)	(-13.2)	(-39.2)	

SI Tab. SII. **Comparison of crystal structures calculated using various theoretical methods.** The PBE-TS structure is called as theoretical structure in the main text.

Table SII summarizes the structure parameters of crystal structures calculated using various theoretical methods. The table also shows the relative deviations between the calculated and experimental structure parameters and $RMSD_{20}$. For explanations of θ_{tor}, θ_{her}, and $RMSD_{20}$ in the table, please refer to the main manuscript. The calculations with the PBE-Grimme scheme, which employs Grimme's dispersion correction [3], and with the PW91-OBS scheme, which employs the generalized gradient approximation of Perdew-Wang 91 [4] and Ortmann-Bechstedt-Schmidt dispersion correction [5], were executed using the software CASTEP. The settings of the cutoff energy for the plane wave, total energy convergence tolerance, and Brillouin zone integration were same as those used for the calculation with PBE-TS. In Table SII, the crystal structure calculated using DFT-D with the PBE-TS scheme best matches the experimental structure, with an $RMSD_{20}$ value of 0.584 Å. The PBE-Grimme and PW91-OBS calculations provide low reproducibility of the experimental structure compared with the PBE-TS calculation due to the difference in the generalized gradient approximation and the dispersion correction schemes; $RMSD_{20}$ values of 1.403 and 2.464 Å were obtained, respectively. The crystal structure calculated with MMFF94 potential has an $RMSD_{20}$ of 1.123 Å; the classical calculation thus shows good accuracy. However, the results of $\Delta\theta_{her}$ indicate that unlike PBE calculations, the MMFF94 potential, which

consists of distance-dependent functions for intermolecular interactions [6], has difficulty reproducing the herringbone arrangement of π-conjugated cores in the C_{10}–DNBDT molecule.

2. Crystal-structure-search methodology

In the crystal calculation, the crystal energy, E_{crystal}, is defined as

$$E_{\text{crystal}} = E_{\text{intra}} + E_{\text{lattice}}, \quad (1)$$

where E_{intra} is the sum of the intramolecular interaction energies for the molecule in the asymmetric unit, and E_{lattice} is a lattice energy, defined as

$$E_{\text{lattice}} = E_{\text{inter}}^{\text{AU}} + \frac{1}{2}\sum_{i}^{N}\sum_{S}^{M}\sum_{J}^{N} E_{\text{inter}}(i; S, J). \quad (2)$$

Here, E_{inter}^{AU} is the sum of the intermolecular interaction energies in the asymmetric unit. Its value is zero because the number of molecules in the asymmetric unit is one in this work. $E_{\text{inter}}(i; S, J)$ is the interatomic interaction energy between atom i in the asymmetric unit and atom J in a symmetry-related unit S, which is generated by applying a symmetry operation to the asymmetric unit. N is the number of atoms in the asymmetric unit, and M is the total number of symmetry-related units within a cut-off radius R_{crystal} from the asymmetric unit; that is, only the molecules for which the closest interatomic distance from the molecule in the asymmetric unit is less than or equal to R_{crystal} are included in the interatomic energy calculations. R_{crystal} was set to 20 Å in this work.

In the generation of initial crystal structures, the space groups are selected according to space group frequency ranking using the Cambridge Structural Database (CSD) [7]. In the ranking, the top 13 space groups are $P2_1/c$, $P\overline{1}$, $C2/c$, $P2_12_12_1$, $P2_1$, $Pbca$, $Pna2_1$, $Pnma$, Cc, $P1$, $C2$, $Pbcn$, and $Pca2_1$; the sum of their frequencies in the CSD is about 90%. Therefore, under the reasonable assumption that C_{10}–DNBDT has a conformation with inversion symmetry in its crystal structure, we selected the maximal subgroups with high frequency and without inversion symmetry of $P2_1/c$, $P\overline{1}$, $C2/c$, $Pbca$, $Pnma$, and $Pbcn$; that is, $P1$, $P2_1$, $C2$, Pc, Cc, $P2_12_12$, $P2_12_12_1$, $Pca2_1$, and $Pna2_1$. The space groups without inversion symmetry among the 13 space groups are included in the selected space groups.

Lattice constant parameters can be determined by a size of asymmetric unit and a type of space group, since the asymmetric unit in the unit cell can be assumed as a space occupied

by one of equivalent units in the specified space group. We define the asymmetric unit as a cuboid block, and a size of the cuboid block is determined by the size of oriented molecule along x, y, and z axes. Initial lattice lengths are calculated by the size of the cuboid block and by the definitions of asymmetric unit predetermined in the specified space group. For example, if we use $Pna2_1$ space group, the asymmetric unit in the unit cell is defined by $0 \leq x \leq 1/4$, $0 \leq y \leq 1$, and $0 \leq z \leq 1$. Therefore, when lengths of three sides of the cuboid block are defined as l_a, l_b, and l_c, initial lattice length a, b, and c are $l_a \times 4$, $l_b \times 1$, and $l_c \times 1$, respectively, and initial lattice angles set to 90 degrees. When there is more than one way of defining the asymmetric unit in the specified space group, we attempt all definitions of asymmetric unit in the generation of initial crystal structures. For example, if we use $Pna2_1$ space group, four ways of defining the asymmetric unit are used: 1: $0 \leq x \leq 1/4$, $0 \leq y \leq 1$, and $0 \leq z \leq 1$, 2: $0 \leq x \leq 1$, $0 \leq y \leq 1/4$, and $0 \leq z \leq 1$, 3: $0 \leq x \leq 1/2$, $0 \leq y \leq 1/2$, and $0 \leq z \leq 1$, and 4: $0 \leq x \leq 1/2$, $0 \leq y \leq 1$, and $0 \leq z \leq 1/2$. The number of definitions of asymmetric unit for the $P1$, $P2_1$, $C2$, Pc, Cc, $P2_12_12$, $P2_12_12_1$, $Pca2_1$, and $Pna2_1$ are 1, 3, 4, 2, 4, 5, 6, 3, and 4, respectively. The oriented molecule is initially positioned at the center of each area of asymmetric unit.

The all initial crystal structures are subjected to a stepwise optimization as follows. The optimization of unit cell dimensions and molecular translation is firstly applied to the initial structures in order to make higher density molecular packing under maintaining the given unique molecular orientations and conformations. After that, the optimized intermediate structures are subjected to the optimization for relaxing all degrees of freedom for representing the crystal structure, that is, intramolecular geometry, molecular orientation and translation, and unit cell dimensions by minimizing the crystal energy, E_{crystal}. These optimizations are performed under the specified space group symmetry. For all optimized crystal structures, the root-mean-square deviation of gradients over all parameters converged to better than 1.0×10^{-5} kcal/mol/Å and the root-mean-square deviation of displacements of the parameters converged to better than 1.0×10^{-6} Å. Adequate space groups and lattice constant parameters of the optimized crystal structures are determined using the software PLATON [8] or Materials Studio 2017 [9]. Duplicated structures are checked using Crystal Packing Similarity Tool [13] in the CSD Python API.

In the determination of final ranking, the crystal structures are assessed based on their calculated energies, $\Delta E_{\mathrm{crystal}}$, and PXRD pattern similarities, ΔS_{PXRD}. The factor α in the equation of A_{crystal} is included to estimate the structural discrepancy as an energetic penalty in the assessment and to control the effect of PXRD pattern similarity on the final ranking. In the present work, α is set to 25 kcal/mol, and the structural discrepancy defined by a ΔS_{PXRD} value of 0.2 is estimated as the energetic penalty of 5.0 kcal/mol because a R-factor of 0.2 is considered a criterion for a correct structure in experimental crystal structure analysis for complicated molecules such as proteins [10].

S_{PXRD} is calculated using Gelder's method [11] with a parameter l of 2.0 degrees. The parameter l defines the width of a relative shift between patterns for calculating the similarity [11]. The 2θ range of 1.0 to 40.0 is used for calculating S_{PXRD}. $S_{\mathrm{PXRD}} = 1$ means that the calculated PXRD pattern completely matches the experimental pattern. The PXRD patterns of the optimized crystal structures are calculated for a wavelength of 1.08 Å. The background in the experimental PXRD data is removed using the software DASH [12] before similarities are calculated.

3. Transport calculation methodology

For charge transport with coupling between charge carriers and molecular vibrations, we calculate the mobility μ_x of a charge carrier along the x direction by using Kubo's linear response theory [14, 15],

$$\mu_x = q \lim_{t\to+\infty} \int_0^t ds \int_0^\beta d\lambda \; \mathrm{Tr}\left\{\hat{\rho}\hat{v}_x(-i\hbar\lambda)\hat{v}_x(s)\right\}. \tag{3}$$

Here, q is the elementary charge of a carrier. The density operator is defined as $\hat{\rho} \equiv \exp(-\beta\hat{H}_e)/Tr\{\exp(-\beta\hat{H}_e)\}$ using the electron Hamiltonian $\hat{H}_e$ and the inverse temperature $\beta \equiv 1/(k_B T)$. The velocity operator at time s in the Heisenberg representation is given by $\hat{v}_x(s) \equiv \hat{U}^\dagger(s)\hat{v}_x\hat{U}(s)$, where $i\hbar\hat{v}_x \equiv [\hat{H}_e, \hat{x}]$ and $\hat{U}(s)$ is the time-evolution operator from initial time 0 to s. To reduce the calculation cost, we compute the time evolution operator numerically using Chebyshev polynomials.

The Hamiltonian of an electron coupled with molecular vibrations is written as $\hat{H}_e(t) = \sum_{N,M} t_{NM}(t)\left(\hat{a}_N^\dagger\hat{a}_M + \hat{a}_M^\dagger\hat{a}_N\right) + \sum_N \varepsilon_N\hat{a}_N^\dagger\hat{a}_N$, where ε_N represents the energy level of the Nth molecular orbital. The effective transfer integrals that include interactions between electron and molecular vibrations are given by the time-dependent form,

$$t_{NM}(t) = \alpha^{intra}\{t_{NM}^0 + \alpha_{NM}^{inter}(t)\}, \tag{4}$$

$$\alpha_{NM}^{inter}(t) = \sum_{l,\mathbf{q}} \Delta t_{NMl\mathbf{q}}^{inter} \sin(\omega_{l\mathbf{q}}^{inter} t + \mathbf{q}\cdot\mathbf{R}_\xi + \phi_{l\mathbf{q}}), \tag{5}$$

where t^0 represents the bare transfer integral. The effects of intermolecular vibrations on the transfer integrals are introduced as the dynamic disorder $\alpha_{NM}^{inter}(t)$, which is given by the superposition of a harmonic oscillation form containing the frequency $\omega_{l\mathbf{q}}^{inter}$ of the lth mode, wave number $\mathbf{q}$, and initial random phase $\phi_{l\mathbf{q}}$ in Eq. (5). The fluctuating amplitude induced by the molecular vibrations is given by $\Delta t_{NMl\mathbf{q}}^{inter} = (\partial t_{NM}^0/\partial\mathbf{u}^{l\mathbf{q}})\Delta\mathbf{r}^{l\mathbf{q}}$, where a component of $\mathbf{u}^{l\mathbf{q}}$ with respect to the nth atom is defined as $\mathbf{e}_n^{l\mathbf{q}}/\sqrt{m_n}$ using the eigenvectors $\mathbf{e}^{l\mathbf{q}}$ of the dynamical matrix and mass m_n of the nth atom. The quantity $(\partial t_{NM}^0/\partial\mathbf{u}^{l\mathbf{q}})$ corresponds to the magnitude of electron-vibration coupling. The amplitude of the displacement is given by $\Delta\mathbf{r}^{l\mathbf{q}} = \sqrt{\hbar n_{l\mathbf{q}}/2\omega_{l\mathbf{q}}}\mathbf{u}^{l\mathbf{q}}$, where $n_{l\mathbf{q}}$ is the number of phonons excited at temperature T. The fast intramolecular vibrations renormalize the bare transfer integral via small polaron formation. The renormalization factor is obtained as

$\alpha^{intra} = \exp\{-(\lambda/2\hbar\omega^{intra})\coth(\beta\hbar\omega^{intra}/2)\}$, where λ and ω^{intra} represent the reorganization energy and the frequency of the most significant contributed normal mode, respectively. [16]

We extract the intermolecular transfer integrals t^0_{NM} by calculating maximally localized Wannier functions (MLWFs) centered on a site $\mathbf{R}_N$ as follows:
$W_{\alpha\mathbf{R}_N}(\mathbf{r}) = \frac{V}{(2\pi)^3}\int_{BZ}\left\{\sum_\beta U^{(\mathbf{k})}_{\alpha\beta}\psi_{\beta\mathbf{k}}(\mathbf{r})\right\}e^{-i\mathbf{k}\mathbf{R}_N}d^3k$, where V is the volume of the unit cell and the Bloch states $\psi_{\alpha\mathbf{k}}(\mathbf{r})$ with band index α and wave vector $\mathbf{k}$ are obtained using DFT-D [17] for the Kohn-Sham Hamiltonian $\hat{H}_{KS}$. The unitary matrix $\mathbf{U}^{(\mathbf{k})}$ is determined so that it minimizes the spread function $S = \sum_\alpha\left\{\langle W_{\alpha\mathbf{0}}|r^2|W_{\alpha\mathbf{0}}\rangle - |\langle W_{\alpha\mathbf{0}}|\mathbf{r}|W_{\alpha\mathbf{0}}\rangle|^2\right\}$. [18] Since the MLWF is located on each molecule, the intermolecular transfer integrals are obtained as $t^0_{NM} = \langle W_{\alpha\mathbf{R}_N}|\hat{H}_{KS}|W_{\alpha\mathbf{R}_M}\rangle$. [19] As an example, we show the calculated MLWF generated from the HOMO band states in the experimental structure of C_{10}–DNBDT in Fig. S4. To reduce the computational cost, we assumed that $(\partial t^0_{NM}/\partial\mathbf{u}^{l\mathbf{q}})$ is independent of $\mathbf{q}$, namely, equal to $(\partial t^0_{NM}/\partial\mathbf{u}^{l\mathbf{q}=\mathbf{0}})$. This is a reasonable assumption because the phonon bandwidth is much narrower than the HOMO bandwidth. Furthermore, we numerically evaluate the quantities $(\partial t^0_{NM}/\partial\mathbf{u}^{l\mathbf{q}=\mathbf{0}})$ using the low-cost dimer method, instead of the high-accuracy but high-cost Wannier method, as used in our previous work. [15]

To reduce the computational cost, we evaluate the intermolecular vibrational modes, $\omega_{l\mathbf{q}}$ and $\mathbf{u}^{l\mathbf{q}}$, based on force field calculations using the software CONFLEX [25]. It is because phonon-band-structure (inter-molecular vibration) calculations using DFT with dispersion correction requires high computational cost. To the best of our knowledge, the calculation examples using DFT are very few and restricted to single crystal of small molecule such as naphthalene [20] and rubrene [21]. Most of practical organic molecules, such as C_8-BTBT, C_{10}-DNTT, and C_{10}-DNBDT discussed in this paper, are large molecule than naphthalene and rubrene because these have long alkyl-side chains to improve solubility. In case of naphthalene single crystal (ID: NAPHTHA31 in Cambridge Structural Database), three inter-molecular optical modes are obtained as 68, 98, and 174cm^{-1} at Γ point by our calculations using force field MMFF94, while these calculated by DFT with vdW-DF functional are given as 53, 70, and 97cm^{-1} [20]. Our calculated frequencies are overestimated, but the differences do not give serious impact to finally obtained mobilities around room temperature.

To calculate the renormalization factor α^{intra}, we need the value of $\hbar\omega^{intra}$. In case of

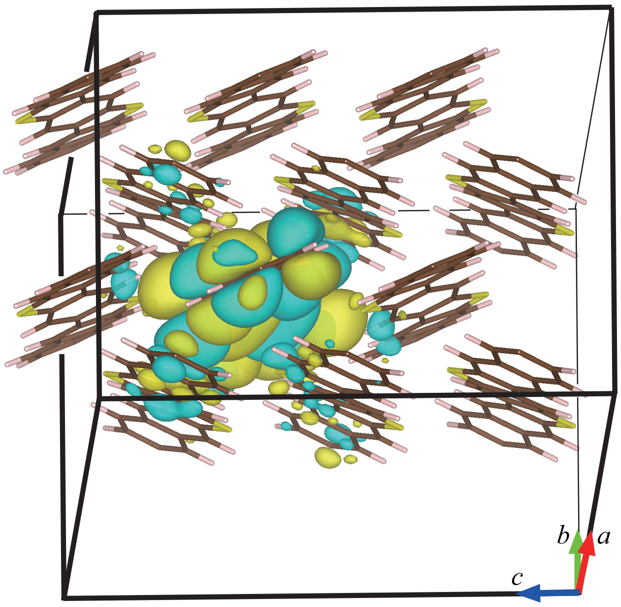

SI Fig. S4. **Experimental structure of a single crystal of C_{10}-DNBDT with the calculated MLWF generated from the HOMO band states in a $1 \times 2 \times 3$ supercell.** For visibility, the alkyl side chains are omitted.

small molecular organic semiconductors, previous work [22] clarified that $\hbar\omega^{intra}$ corresponds to Carbon-Carbon bond stretching mode. The frequency takes value ranging from 0.15 to 0.20 eV depending on molecules. From a viewpoint of computational cost, we employ 0.15 eV as the typical value of $\hbar\omega^{intra}$ since the difference of calculated $\hbar\omega^{intra}$ to 0.15eV does not seriously affect calculated mobility around room temperature. In our previous work [15], we confirmed that the calculated mobilities using this typical value for several organic semiconductors show good agreement with experimental values. The reorganization energy λ of a C_{10}–DNBDT molecule is obtained as 91 meV using the adiabatic potential energy surface method [23] with the B3LYP/6-31G(d) level derived using the software GAMESS [24].

4. Computational resources and times

Calculations	Computational resources	Elapsed time (days)
Crystal structure search (ii)	Intel Xeon E5-2680 v2 (2.80 GHz), 200 cores	22
Re-optimization by DFT-D (iii)	Intel Xeon E5-2690 (2.90 GHz), 8 cores	15
Transport property (iv)		
Calc. of t^0_{NM}	Intel Xeon E5-2670 (2.60 GHz), 26 cores	0.5
Calc. of $\partial t^0_{NM}/\partial \mathbf{u}^{l\mathbf{0}}$	COMA (PACS-IX), 20 cores per each (N, M, l)	1
Calc. of μ	Intel Xeon E5-2697 v4 (2.30GHz), 80 cores	3

SI Tab. SIII. **Summary of computational resources and times for the proposed method.** (ii)–(iv) correspond to the steps in Fig. 1 of the main text.

The computational resources and times for steps (ii) to (iv) in Fig. 1 are summarized in Table SIII. Here, the computational time for the conformational search in step (i) is negligible; it is only 3 minutes using parallel computing with 20 cores of an Intel Xeon E5-2680 v2 CPU. The computational time for step (ii) is 22 days using the TUT supercomputer system, and that for step (iii) is 15 days using a general workstation. The intermolecular transfer integrals t^0_{NM} are calculated using the Wannier method based on DFT-D [17, 18]. $\partial t^0_{NM}/\partial \mathbf{u}^{l\mathbf{0}}$ are obtained using the low-cost dimer method. The calculation time for $\partial t^0_{NM}/\partial \mathbf{u}^{l\mathbf{0}}$ for each (N, M, l) is about 1.9 hours using the supercomputer COMA (PACS-IX) at the University of Tsukuba. The numbers of (N, M) pairs and intermolecular vibrational modes l are 6 and 210, respectively. The computational time for all $\partial t^0_{NM}/\partial \mathbf{u}^{l\mathbf{0}}$ is about 1 day using the supercomputer COMA. We employed a monolayer consisting of 200×200 unit cells to obtain the mobility. The wave packet dynamics are computed up to 2 ps with a time step of 0.5 fs.

[1] Izawa, T., Miyazaki, E. & Takimiya, K. Molecular Ordering of High-Performance Soluble Molecular Semiconductors and Re-evaluation of Their Field-Effect Transistor Characteristics. *Advanced Materials* **20**, 3388–3392 (2008).

[2] Gao, P. et al. Benzo[1,2-b:4,5-b']bis[b]benzothiophene as solution processible organic semiconductor for field-effect transistors. *Chem. Commun.*, 1548–1550 (2008).

[3] Grimme, S., Semiempirical GGA–type density functional constructed with a long–range dispersion correction. *J. Comput. Chem.*, **27**, 1787–1799 (2006).

[4] Perdew, J. P., Chevary, J. A., Vosko, S. H., Jackson, K. A., Pederson, M. R., Singh, D. J., & Fiolhais, C. Atoms, molecules, solids, and surfaces: Applications of the generalized gradient approximation for exchange and correlation, *Phys. Rev. B* **46**, 6671–6687 (1992).

[5] Ortmann, F., Bechstedt, F., & Schmidt, W. G., Semiempirical van der Waals correction to the density functional description of solids and molecular structures. *Phys. Rev. B.* **73**, 205101 (2006).

[6] Halgren, T. A. Merck Molecular Force Field. II. MMFF94 van der Waals and Electrostatic Parameters for Intermolecular Interactions. *J. Comput. Chem.*, **17**, 520–552 (1996).

[7] CSD Space Group Statistics, The Cambridge Crystallographic Data Centre, 12 Union Road, Cambridge, CB2 1EZ, United Kingdom, http://www.ccdc.cam.ac.uk (2018).

[8] Spek, A. L. Structure validation in chemical crystallography. *Acta Cryst.* D**65**, 148–155 (2009).

[9] Dassault Systemes BIOVIA, Visualizer, 2017R2, San Diego: *Dassault Systemes*, 2017.

[10] Rondeau, J.-M., Schreuder, H., Protein Crystallography and Drug Discovery, *The Practice of Medicinal Chemistry (Fourth Edition)*, 511–537 (2015).

[11] De Gelder, R., Wehrens, R. & Hageman, J. A. A generalized expression for the similarity of spectra: Application to powder diffraction pattern classification. *J. Comput. Chem.* **22**, 273–289 (2001).

[12] David, W. I. F., Shankland, K., van de Streek, J., Pidcock, E., Motherwell, W. D. S., Cole, J. C. DASH: a program for crystal structure determination from powder diffraction data. *J. Appl. Cryst.* **39**, 910–915 (2006).

[13] Motherwell, S., Chisholm, J. A. COMPACK: a program for identifying crystal structure similarity using distances. *J. Appl. Crystallogr.* **38**, 228–231 (2005).

[14] Kubo, R. Statistical-Mechanical Theory of Irreversible Processes. I. General Theory and Simple Applications to Magnetic and Conduction Problems. *J. Phys. Soc. Jpn.* **12**, 570–586 (1957).

[15] Ishii, H., Inoue, J., Kobayashi, N., & Hirose, K. Quantitative mobility evaluation of organic semiconductors using quantum dynamics based on density functional theory. *Phys. Rev. B* **98**, 235422 (2018).

[16] Houili, H., Picon, J. D., Zuppiroli, L. & Bussac, M. N. Polarization effects in the channel of an organic field-effect transistor. *J. Appl. Phys.* **100**, 023702 (2006).

[17] Giannozzi, P. et al., Quantum ESPRESSO: a modular and open-source software project for quantum simulations of materials. *J. Phys.: Condens. Matter.* **21**, 395502 (2009).

[18] Mostofi, A. A. et al., wannier90: A Tool for obtaining maximally-localised Wannier functions. *Comput. Phys. Commun.* **178**, 685–699 (2008).

[19] Ishii, H., Kobayashi, N. & Hirose, K. Charge transport calculations by a wave-packet dynamical approach using maximally localized Wannier functions based on density functional theory: Application to high-mobility organic semiconductors. *Phys. Rev. B* **95**, 035433 (2017).

[20] Brown-Altvater, F., Rangel, T., & Neaton, J. B., *Ab initio* phonon dispersion in crystalline naphthalene using van der Waals density functionals. *Phys. Rev. B* **93**, 195206 (2016).

[21] Xie, X., Santana-Bonilla, A., & Troisi, A., Nonlocal Electron-Phonon Coupling in Prototypical Molecular Semiconductors from First Principles. *J. Chem. Theory Comput.* **14**, 3752–3762 (2018).

[22] Girlando, A., Grisanti, L., Masino, M., Brillante, A., Della Valle, R. G., & Venuti, E., Peierls and Holstein carrier-phonon coupling in crystalline rubrene. *J. Chem. Phys.* **135**, 084701 (2011).

[23] Deng, W.-Q. & Goddard III, W. A. Predictions of Hole Mobilities in Oligoacene Organic Semiconductors from Quantum Mechanical Calculations. *J. Phys. Chem. B* **108**, 8614–8621 (2004).

[24] Schmidt, M. W. et al. General Atomic and Molecular Electronic Structure System. *J. Comput. Chem.* **14**, 1347–1363 (1993).

[25] Goto, H. Obata, S. Nakayama, N. and Ohta, K. CONFLEX8, Conflex, Tokyo, Japan (2017).
